# Video-urodynamic characteristics of non-neurogenic, idiopathic underactive bladder in men – A comparison of men with normal tracing and bladder outlet obstruction

**DOI:** 10.1371/journal.pone.0174593

**Published:** 2017-04-04

**Authors:** Yuan-Hong Jiang, Hann-Chorng Kuo

**Affiliations:** Department of Urology, Buddhist Tzu Chi General Hospital and Tzu Chi University, Hualien, Taiwan; University of Oklahoma Health Sciences Center, UNITED STATES

## Abstract

**Objective:**

Underactive bladder is frequently encountered in elderly patients. It may result from detrusor underactivity (DU) or low detrusor contractility due to a urethral sphincter inhibitory effect. This study analyzed the video-urodynamic study (VUDS) characteristics of patients with underactive bladder in a large cohort of men with lower urinary tract symptoms (LUTS).

**Methods:**

Male patients with LUTS who had failed the initial treatment were consecutively enrolled. All patients underwent detailed urological investigations including prostate measurement, free uroflowmetry, post-void residual volume (PVR) measurement, cystoscopy and VUDS. The VUDS characteristics of the men with underactive bladder were analyzed and compared with those of men with bladder outlet obstruction and normal tracing.

**Results:**

A total of 1329 men who underwent VUDS were included in this retrospective analysis. After VUDS, the final diagnosis was DU in 165 patients, poor relaxation of external sphincter (PRES) in 525, bladder outlet obstruction in 501, and normal tracing in 138. VUDS findings in DU patients showed a slowly increased detrusor pressure, intermittent detrusor contractions, or early decline of detrusor contraction, resulting in a low maximum flow rate (Qmax), and large PVR. In comparison with the PRES groups, DU patients were older, had reduced bladder sensation, lower detrusor pressure (Pdet), lower Qmax, larger PVR volume, and lower voiding efficiency. Patients with urodynamic PRES also had low-pressure–low-flow tracings, but their bladder sensation was similar to that with normal tracing. DU patients with very low Pdet also had low detrusor tonicity, and more medical co-morbidities than the other groups did.

**Conclusion:**

Idiopathic underactive bladder in elderly men could be attributed to urodynamic DU and PRES. DU is associated with old age, reduced bladder sensation, low voiding efficiency, and medical co-morbidities.

## Introduction

Underactive bladder is frequently encountered in elderly patients with chronic medical or neurological diseases. Underactive bladder causes lower urinary tract symptoms (LUTS) of hesitancy, slow stream, dysuria, and incomplete voiding. Patients with underactive bladder might have low detrusor contractility or a non-contractile detrusor; both are known as forms of detrusor underactivity (DU) in urodynamic terminology. In patients with DU, chronic urinary retention or large post-void residual (PVR) volume is frequently noted, which is usually difficult to manage. The pathophysiology of underactive bladder may involve neurogenic, myogenic, and bladder outlet pathologies [1]. Recent studies also reveal that urothelial dysfunction of the urinary bladder may be associated with impaired bladder sensation as well as impaired detrusor contractility [2].

DU is a urodynamic term that is defined by the International Continence Society as a contraction of reduced strength and/or duration, resulting in prolonged bladder emptying and/or failure to achieve complete bladder emptying within a reasonable time [3]. Clinically, patients with DU usually have diminished bladder fullness or urgency sensation and cannot contract the detrusor sufficiently to complete bladder emptying. The urodynamic features of underactive bladder include a non-contractile detrusor, low bladder pressure, or poorly sustained detrusor contraction in association with a poor flow rate with or without a large PVR [4]. Some of the patients with underactive bladder may have both detrusor hyperactivity and inadequate contractility, resulting in urgency incontinence and large PVR [5].

The pathogenesis of underactive bladder is likely to be multifactorial [1]. The causes of underactive bladder or DU include diabetes mellitus, bladder outlet obstruction (BOO), aging, neurological diseases, spinal cord lesions, and pelvic plexus and infectious neurologic problems [6]. DU can result from damage to the bladder afferent pathways, bladder efferent pathways, lumbosacral spinal cord, or pure detrusor failure [7].

In young adult men, underactive bladder may also result from weak detrusor contractility without neuropathy or anatomical BOO. Low-pressure–low-flow urodynamic tracings are the characteristic findings in this group of patients [8]. The low voiding detrusor pressure may or may not be associated with low bladder emptying efficiency [9]. Poor relaxation of external urethral sphincter (PRES) is proposed to play an important role in the development of underactive bladder in younger men [10]. Recently, urologists have had an interest in exploring the pathophysiology of underactive bladder and have sought to investigate new treatments for this common lower urinary tract dysfunction. We analyzed the videourodynamic study (VUDS) characteristics of a large cohort of men with underactive bladder and LUTS and compared their data with patients with normal pressure flow tracing and anatomical BOO.

## Materials and methods

Men with persistent LUTS who had failed the initial medical treatment were consecutively enrolled to undergo VUDS to obtain a better differential diagnosis of their vesicourethral dysfunction. The patients with overt clinical diagnosis of BOO due to a large prostate and large PVR, acute urinary retention, overt neuropathy such as stroke, Parkinson’s disease, spinal cord injury, presence of bladder stones, and acute urinary tract infection were excluded.

This study was approved by the Institution Review Board of Buddhist Tzu Chi General Hospital (IRB 103-145-A). Informed consent was waived by the committee due to the retrospective nature of the study. All patients had received detailed urological investigations including transrectal sonography of the prostate for total prostatic volume and transition zone index, free uroflowmetry, PVR volume measurement, cystoscopy, and VUDS. The medical diseases that were still under treatment were also recorded.

VUDS was performed according to the recommendations of the International Continence Society [3]. The details of the studies are described and reported in detail in a previous study [11]. The urodynamic parameters of first sensation of filling, full sensation, cystometric bladder capacity, bladder compliance, maximum flow rate (Qmax), PVR, voiding detrusor pressure at Qmax (P_det.Qmax_), bladder outlet obstruction index (BOOI, defined as Pdet.Qmax—[2×Qmax]; bladder contractility index, (BCI, defined as P_det.Qmax_ + 5 x Qmax), and voiding efficiency (VE, defined as voided volume/bladder capacity x 100) were measured and recorded.

The VUDS was repeated at least twice to obtain reproducible pressure flow tracings. BOO, bladder neck dysfunction (BND), PRES, urethral sphincter dyssynergia, increased bladder sensation, detrusor overactivity, DU, and detrusor hyperactivity and inadequate contractility were diagnosed according to the findings of characteristic bladder dysfunction and bladder outlet dysfunction during the study [11]. In VUDS, patients with DU might have a good external sphincter electromyographic (EMG) relaxation during voiding but the P_det.Qmax_ and Qmax were low. However, some DU patients might have a tight bladder neck or a poor relaxing urethral sphincter in association with a low Pdet and low Qmax.

The definition of BOO was based on the provisional International Continence Society definition of obstruction [3]. BOO is defined as a pressure-flow study showing a P_det.Qmax_ greater or equal to 50 cmH_2_O or a BOOI greater or equal to 40. In patients with pressure flow results not reaching the criteria of BOO, prostatic obstruction and BND was diagnosed based on the narrow site in the videourodnamic image, regardless of whether the voiding P_det.Qmax_ was high or normal [11,12].

PRES was diagnosed based on (1) low voiding P_det.Qmax_, (2) non-relaxation of urethral sphincter EMG, and (3) videourodynamic image showing narrow membranous urethral during voiding [13]. If urethral sphincter EMG showed hyperactivity in combination with a high voiding pressure, urethral sphincter dyssynergia was diagnosed. The Qmax during VUDS was compared with the free uroflowmetry before the study. If the Qmax at VUDS was lower than that of free uroflowmetry, the Qmax at free uroflowmetry was adapted for analysis in this study.

After the VUDS, the men with bladder hypersensitivity, detrusor overactivity, detrusor hyperactivity and inadequate contractility were excluded from the final analysis. The patients with a low voiding pressure and low flow rate with or without a large PVR were grouped as having underactive bladder. The prostatic measurements and urodynamic parameters were compared among patients with underactive bladder. The men with normal videourodynamic tracing and with BOO were selected for the comparative study. The age and urodynamic parameters were compared among patients with normal tracing, DU, PRES, and BOO. Furthermore, patients with DU were grouped as low voiding pressure (Pdet ≥5 cmH_2_O) and very low voiding pressure (Pdet <5 cmH_2_O) and the urodynamic parameters were compared between groups.

The patients with DU and VUDS proven BND or prostatic obstruction due to small prostatic volume (< 30ml) and refractory to drug treatment received transurethral incision of prostate (TUIP). Transurethral resection of the prostate (TURP) was performed in patients with prostatic obstruction due to large prostatic volume (≥ 30ml). Medical treatment included alpha-adrenergic blocker and baclofen, or onabotulinumtoxinA urethral sphincter injection for patients with DU and tight urethral sphincter during VUDS. Patients who did not resume spontaneous voiding or had large PVR were managed with voiding training by trocar cystostomy or clean intermittent catheterization.

### Statistical analysis

Continuous variables are presented as means ± standard deviations, and categorical data are presented as number and percentage (%). Statistical comparisons between the groups were tested using the chi-square test for categorical variables, and the Wilcoxon rank-sum test for continuous variables. Statistical assessments were considered significant when *p* was < 0.05. Statistical analyses were performed using SPSS 15.0 statistical software (SPSS Inc., Chicago, IL).

## Results

In this retrospective study, 1329 men who had undergone VUDS were included. After VUDS, the final diagnosis was DU in 165 men, PRES in 525, BOO in 501, and normal VUDS tracing in 138 men. The BOO group included patients with benign prostatic obstruction (n = 205/501, 40.9%), BND (n = 283/501, 56.5%), and urethral stricture (n = 13/501, 2.6%).

The mean age of the men in these four groups is listed in Table 1. The mean age of the men with DU (71.1 ± 11.7 years) was significantly older than that in the normal and PRES groups. Among the men with DU, 41.2% of them were older than 75 years, 30.7% were 65 to 74 years old, and 28.1% were less than 64 years old. Total prostatic volume was the largest in the patients with BOO, patients with DU ranked second (37.6 ± 23.7 ml), but the mean total prostatic volume was not significantly greater than in the other groups.

**Table 1:** Video urodynamic parameters in men with underactive bladder.

	Normal	DU	PRES	BOO	ANOVA
	(n = 138)	(n = 165)	(n = 525)	(n = 501)	
Age (years)	62.9±10.2	71.1±11.7 *	65.3±11.3	65.6±10.5	<0.0001
TPV (ml)	30.1±13.6	37.6±23.7	29.5±15.8	61.5±41.9	<0.0001
TZI (%)	32.7±12.1	30.1±12.5	42.6±14.8	47.5±12.8	<0.0001
Urodynamic DO (%)	0	0	5.7%	77.3%	<0.0001
FSF (ml)	171±78.9	214±117 *	148±71.2	113±58.0	<0.0001
FS (ml)	299±96.2	324±127 *	255±101	179±87.8	<0.0001
CBC (ml)	526±151	409±150 *	348±136	260±131	<0.0001
Compliance	98.5±88.1	73.1±93.3	72.7±68.6	53.5±58.9	<0.0001
Pdet (cmH_2_O)	31.3±9.94	9.74±10.6 *	29.1±22.3	70.1±27.7	<0.0001
Qmax (ml/s)	19.3±4.24	1.92±3.30 *	9.40±4.72	7.79±4.82	<0.0001
PVR (ml)	28.0±65.3	348±188*	69.4±100	70.3±99.2	<0.0001
BCI	128±23.1	19.3±23.6 *	76.0±34.5	105±34.9	<0.0001
VE (%)	95.6±6.28	17.6±31.1 *	79.1±26.9	80.4±26.4	<0.0001
BOOI	-7.23±13.3	5.91±9.39	10.3±23.1	54.6±29.8	<0.0001

* Significant difference between DU and PRES; BCI: bladder contractility index, BOO: bladder outlet obstruction, BOOI: bladder outlet obstruction index, CBC: cystometric bladder capacity, DO: detrusor overactivity, DU: detrusor underactivity, FS: full sensation, FSF: first sensation of filling, Pdet: detrusor pressure, PRES: poor relaxation of external sphincter, Qmax: maximum flow rate, TPV: total prostate volume, TZI: transition zone index, VE: voiding efficiency.

**Fig 1:**
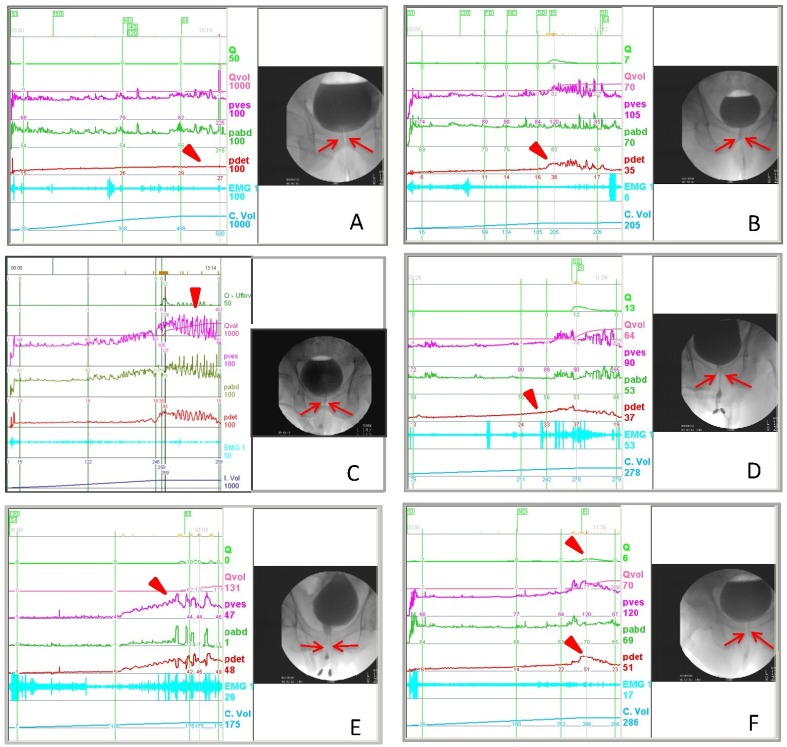
The videourodynamic characteristics of patients with underactive bladder. (A) Detrusor acontractile, reduced bladder sensation, no detrusor contractility (arrow head), and a tight bladder neck (arrows). (B) Low detrusor contractility, low detrusor pressure (arrow heads) and low Qmax, open bladder outlet during voiding (arrows). (C) Low detrusor contractility and early termination, patient used abdominal pressure to empty the bladder (arrow head), the bladder outlet is open (arrows). (D) Detrusor overactivity and impaired contractility, patient had a hypertonic bladder at capacity (arrow head) but the voiding pressure and Qmax are low, the bladder outlet is open (arrows). (E) Slowly increased detrusor pressure (arrow head) and poor relaxing urethral sphincter (arrows). (F) Normal voiding pressure and low Qmax (arrow head), with a narrow bladder neck during voiding (arrows).

VUDS findings in men with DU showed a non-contractile detrusor, slowly increasing detrusor pressure, intermittent detrusor contractions, or early decline of detrusor contractions, resulting in a low Qmax and large PVR. The bladder outlet showed no narrowing of the bladder neck and prostatic urethra. Some DU patients might have a tight bladder neck or a poor relaxing urethral sphincter in association with a low Pdet and low Qmax (Fig 1).

The bladder sensation during VUDS showed that first sensation of filling (214 ± 117 ml) and full sensation (324 ± 127 ml) were significantly greater in men with DU than in the normal tracing and other groups. Cystometric bladder capacity (409 ± 150 ml) in men with DU was significantly greater than that in the PRES and BOO groups but smaller than in the normal tracing group. In addition, patients with DU showed the lowest P_det.Qmax_ (9.74 ± 10.6 cmH_2_O), lowest Qmax (1.92 ± 3.30 ml/s), the largest PVR (348 ± 188 ml/s), and a mean bladder compliance between the normal tracing and BOO groups. When we compared the BCI, BOOI, and VE among the groups, BCI (19.3 ± 23.6) and VE (17.6 ± 31.1%) were the lowest in patients with DU, the BOOI (5.91 ± 9.39) indicated no obstruction in the DU group.

During VUDS, a P_det.Qmax_ of <5 cmH_2_O is nearly equal to detrusor acontractility. It is not easy to identify a detrusor contraction of lower than 5 cmH_2_O. Therefore we chose this value as a cut off point for very low pressure (acontractile) and low pressure (low contractility) and compared the urodynamic parameters between groups. When we compared urodynamic characteristics in DU patients with a very low (<5 cmH_2_O) and low (≥5 cmH_2_O) voiding pressure, there was no significant difference in the bladder sensation, bladder capacity and PVR between the groups. However, the bladder compliance was significantly larger, and the Pdet, Qmax, BCI and VE were significantly lower in patients with very low voiding pressure than in patients with low voiding pressure (Table 2).

**Table 2:** The urodynamic parameters between patients with detrusor underactivity of different detrusor contractility.

	**Pdet.Qmax <5cmH**_**2**_**O**	**Pdet.Qmax ≥5cmH**_**2**_**O**	**P value**
	(n = 54)	(n = 111)	
FSF (ml)	225 ± 117	209 ± 117	0.409
FS (ml)	340 ± 130	317 ± 126	0.276
CBC (ml)	420 ± 140	405 ± 155	0.540
Compliance	110 ± 118	55.5 ± 73.7	0.003
Pdet (cmH_2_O)	0.91 ± 1.21	14.0 ± 10.5	<0.0001
Qmax (ml/s)	0.83 ± 2.42	2.43 ± 3.56	0.001
PVR (ml)	397 ± 169	327 ± 193	0.023
BCI	5.06 ± 12.3	26.1 ± 24.7	<0.0001
VE (%)	8.0 ± 23.5	22.2 ± 33.3	0.002
BOOI	-0.75 ± 4.80	9.10 ± 9.38	<0.0001

BCI: bladder contractility index, BOOI: bladder outlet obstruction index, CBC: cystometric bladder capacity, FS: full sensation, FSF: first sensation of filling, Pdet: detrusor pressure, Qmax: maximum flow rate, VE: voiding efficiency.

In contrast to the men with DU, those with PRES did not show a significant difference in age (65.3 ± 11.3 years), first sensation of filling (148 ± 71.2 ml), full sensation (255 ± 101 ml), cystometric bladder capacity (348 ± 136 ml), Qmax (9.40 ± 4.72 ml/s), PVR (69.4 ± 100 ml), BCI (76.0 ± 34.5), VE (79.1 ± 26.9%) compared with those of the BOO group. The first sensation of filling in the PRES group was significantly lower than that of the normal tracing men. The VE was significantly lower than that of normal tracing men, but was similar with that of the BOO group. The Pdet (29.1 ± 22.3 cmH_2_O) was significantly lower than BOO group and BOOI (10.3 ± 23.1) also showed no obstruction in patients with PRES. Compared the urodynamic parameters of patients with PRES and DU, reduced bladder sensation of first sensation of filling, full sensation and cystometric bladder capacity, lower Pdet, lower Qmax, larger PVR, lower BCI and lower VE were significant in DU patients (all p<0.001).

Concerning the medical comorbidities in patients with DU and other groups, our data did not show a significant difference between other lower urinary tract dysfunction groups and the normal tracing group. However, DU patients with very low voiding pressure had significantly more medical comorbidities (mean, 3.5 ± 2.7 medical diseases) than in the PRES (mean, 1.7 ± 1.4) and normal tracing groups (mean, 0.8 ± 1.2).

In 86 patients with DU, surgical or medical treatment was given to facilitate spontaneous voiding. Among all 86 male patients, aged from 44 to 90 (mean 73) years, 34 (39.5%) had acute or chronic urinary retention and 52 (60.5%) could void with large PVR. Satisfactory outcome (VE >50%) was achieved in 63 (72.7%) patients, including 38 (86.4%) out of 44 patients who received TURP, 11 (68.8%) of 16 who received TUIP, 9 (47.4%) of 19 who received medical treatment alone and 5 (71.4%) of 7 who received urethral sphincter botulinum toxin A injection.

## Discussion

This study demonstrates that low-pressure–low-flow underactive bladder involves urodynamic DU and PRES. Both lower urinary tract dysfunctions result in low Qmax, increased PVR and LUTS including slow stream and dysuria. However, the urodynamic characteristics of DU and PRES differ for bladder sensation, voiding pressure, and VE. Urodynamic DU is associated with reduced bladder sensation and a larger cystometric bladder capacity than PRES or BOO. These urodynamic characteristics may help urologists in the diagnosis of underactive bladder and with decisions on its treatment.

Underactive bladder due to DU is a common urological problem in elderly patients presenting with urinary retention and LUTS. In one investigation, DU was found in nearly two-thirds of the incontinent institutionalized elderly patients [14]. DU is also common in elderly patients with general weakness and medical diseases such as diabetes mellitus, terminal cancer, and after major surgery [15]. In men, chronic BOO can result in detrusor muscle damage or neurological inhibition, which interfere the integration of muscle-mucosal mechanoreceptors, mucosal mechanoreceptors, and chemoreceptors, causing low detrusor contractility or non-contractility [16]. Our study also showed that men with DU and very low voiding pressure were significantly older and had more medical comorbidities than those with BOO or PRES, indicating these patients might have a poor general condition and thus, their detrusor contractility was lower than that in the other groups. In clinical practice, some DU patients have detrusor function recovery after they regain their health. During any period of urinary retention, the bladder should not be allowed to become overdistended to avoid bladder ischemia and further oxidative stress and damage.

BOO results in structural and functional changes to the bladder wall. The detrusor contractility may be reduced in men with chronic urinary retention or large PVR due to benign prostatic hyperplasia with obstruction [17]. However, it is difficult to differentiate idiopathic DU from chronic urinary retention secondary to BOO. Men with chronic BOO might also have a low detrusor pressure and large PVR. In this study, we did not include patients with chronic urinary retention due to BOO to prevent confusion in interpretation of urodynamic variables. The DU and PRES patients in the study all had small prostates, a low BCI, low VE and low BOOI, suggesting that underactive bladder might have resulted from urothelial or myogenic causes rather than BOO.

A normal bladder sensation and transduction of stretch (urothelial pathway) are essential for normal micturition [5]. A normal perception of bladder fullness and urge sensation is the fundamental basis for normal micturition. Patients with a severe cortical degenerative disease may lack bladder perception and be unable to initiate voiding. Aging causes structural and functional changes of the bladder afferent nerves and detrusor power, and reflex activity could be impaired [18]. In our study, patients with DU had reduced bladder first sensation of filling and full sensation with a larger cystometric bladder capacity that those in the PRES or BOO groups, suggesting the afferent innervation in patients with DU could have been damaged regardless of the etiology. In contrast, patients with PRES, although they also presented with low flow and emptying LUTS, their bladder sensation did not significantly differ from that of normal tracing patients or those with BOO. The low flow rate in PRES might not be caused by impaired afferent nerves but could be due to inhibition of detrusor contractility.

Detrusor contractility in DU patients could be low or nearly acontractile. As shown in Table 2, the bladder sensation was similar among the men with very low voiding pressure and low voiding pressure, but the contractility was different, and PVR was significantly greater in patients with very low voiding pressure. Interestingly, the bladder compliance was significantly larger in patients with very low voiding pressure (<5 cmH2O), suggesting that the detrusor tonicity in this patient group was significantly lower than patients with Pdet ≥ 5 cmH_2_O. Loss of detrusor tonicity is associated with a large bladder compliance, very lower voiding pressure and a large PVR. Clinically, we also observed improved detrusor tonicity before regaining contractility in long-term follow-up of patients with DU. The detrusor tonicity (bladder compliance) might also indicate the residual detrusor contractility.

The inhibitory effects of the detrusor contraction by the striated urethral sphincter via the alpha-adrenergic activity may also play a role in the development of underactive bladder. Afferent nerves of the pudendal nerve are postulated to have a potential modulatory effect on sympathetic neuronal control in various neuropathic and non-neuropathic bladder dysfunctions, but the mechanism and pathways remain unknown [19]. A poorly relaxed urethral sphincter is thought to cause increased urethral afferent activity, which inhibits bladder afferent signaling, leading to poor bladder sensation and low detrusor contractility. Therefore, application of sacral neuromodulation in patients with Fowler’s syndrome may restore normal voiding [20]. Patients with stroke and incomplete bladder emptying might also develop DU due to spasticity of the external sphincter [21]. In young men with LUTS, BND, PRES, and DU are the main urodynamic abnormalities [8,22]. Treatment targeting the bladder neck or urethral sphincter hyperactivity could improve normal detrusor contractility and VE in patients without anatomical BOO but with incomplete bladder emptying.

One might question the role of VUDS as compared to the conventional urodynamics on the diagnosis and management of DU. VUDS provides pressure flow parameters and real time voiding cystourethrographic images to assess different vesicourethral dysfunctions, such as BND, BPO, PRES or bladder dysfunction, during bladder filling and empty. In patients with a low detrusor contractility, we can learn the bladder outlet conditions and differentiate BOO from patients with DU; bladder outlet surgery may reverse detrusor contractility or facilitate spontaneous voiding. In this study, part of the patients with DU regained spontaneous voiding and VE after TUIP, TURP, medical treatment or urethral sphincter onabotulinumtoxinA injection further indicated the value of VUDS to identify the not opening site at the bladder neck or external sphincter in clinical management of patients with DU.

The primary strength of this study is its large sample size. The main limitation of the study is the lack of data on the differential diagnosis of DU etiologies. Because patients with DU had multiple medical co-morbidities, the etiologies of DU may mix with each other and difficult to make differentiation.

## Conclusion

Idiopathic underactive bladder may be attributed to urodynamic DU and PRES. DU is associated with older age, reduced bladder sensation, and medical comorbidities. DU patients with very low voiding pressure also have low detrusor tonicity and a large PVR in addition to diminished bladder sensation. VUDS provides information to determine the vesicourethral dysfunction in men with underactive bladder. Videourodynamic data also aid in determining treatment options.
